# Making healthcare work for young people

**DOI:** 10.1136/archdischild-2017-314573

**Published:** 2018-01-05

**Authors:** Janet E McDonagh, Albert Farre, Helena Gleeson, Tim Rapley, Gail Dovey-Pearce, Debbie Reape, Emma Rigby, Allan F Colver, Jeremy R Parr

**Affiliations:** 1 Centre for Musculoskeletal Research, University of Manchester, Manchester, UK; 2 School of Health and Population Sciences, University of Birmingham, Birmingham, UK; 3 Department of Endocrinology, University Hospital Birmingham, Birmingham, UK; 4 Institute of Health and Society, Newcastle University, Newcastle upon Tyne, UK; 5 Northumbria Healthcare NHS Foundation Trust, North Shields, UK; 6 Association for Young People’s Health, London, UK; 7 Institute of Neuroscience, Newcastle University, Newcastle Upon Tyne, UK

**Keywords:** adolescent health, health services research

We would like to report the clinical translation of research reported in this journal regarding implementation of developmentally appropriate healthcare for young people.^1 2^ A National Health Service (NHS) toolkit is now available to support the implementation of developmentally appropriate healthcare in hospitals in the UK and globally.

Adolescent health is a neglected yet pressing global issue affecting the largest generation in human history.^3^ However, increasing knowledge on adolescent and young adult development offers equally unprecedented opportunities to transform traditional models of healthcare delivery to create adolescent-responsive health systems.^3^ In countries such as the UK, where adolescent medicine is not a recognised medical specialty, the routine embedding of *developmentally appropriate healthcare for young people* has the potential to enable system-wide adolescent-responsive healthcare delivery. In the recent National Institute for Health and Care Excellence guidance for transition, it was highlighted that transitional care should be ‘developmentally appropriate’.^4^ However, there is still the need for further guidance on the practical implementation of developmentally appropriate healthcare for young people, as a core element of clinical work that goes beyond the remit of transitional care and articulates the care provided for all young people attending health services. Furthermore, as we have previously reported in this journal, developmentally appropriate healthcare is ill defined in the literature^1^ and not consistently understood by UK-based health practitioners and managers.^2^

In response to our research findings, we developed an NHS toolkit (within a wider programme of research funded by the National Institute for Health Research (see http://research.ncl.ac.uk/transition/). The NHS toolkit gives practical suggestions about (1) how healthcare can be tailored for the young person and multidisciplinary team across an institution and (2) being responsive to young people’s needs as they develop and change during adolescence and young adulthood. The toolkit can be accessed at https://northumbria.nhs.uk/dahtoolkit and is designed to support everyone working in the NHS, from clinicians to chief executives, to promote the health of young people and to play their part in making healthcare work for this particular group. We hope the toolkit will be useful to health professionals as we move towards the NHS becoming a truly adolescent-responsive health system.
